# INITIAL CLINICAL PRESENTATION IN CASES OF INBORN ERRORS OF METABOLISM IN A REFERENCE CHILDREN’S HOSPITAL: STILL A DIAGNOSTIC CHALLENGE

**DOI:** 10.1590/1984-0462/;2017;35;3;00012

**Published:** 2017-07-31

**Authors:** Andressa Romão, Priscila Endlich Alves Simon, José Eduardo Coutinho Góes, Louise Lapagessede Camargo Pinto, Roberto Giugliani, Gisele Rozone de Luca, Francisca Ligia Cirilo Carvalho

**Affiliations:** a Hospital Ministro Costa Cavalcanti, Foz do Iguaçu, PR, Brasil.; b Maternidade Carmela Dutra, Florianópolis, SC, Brasil.; c Hospital Infantil Joana de Gusmão, Florianópolis, SC, Brasil.; d Hospital de Clínicas de Porto Alegre, Porto Alegre, RS, Brasil.

**Keywords:** Inborn errors of metabolism, Clinical symptoms, Diagnosis, Child

## Abstract

**Objective:**

To assess the initial clinical presentation of confirmed cases of inborn errors of metabolism (IEM) at a reference facility for pediatric care.

**Methods:**

Cross-sectional, observational and descriptive study with data collection of outpatients, from January 2009 to December 2013. Inclusion criterion: referral to IEM investigation. Exclusion criterion: prior diagnosis of IEM. Analyzed variables: identification data; status of diagnostic investigation; family history of IEM; initial clinical presentation, laboratory abnormalities related to the hypothesis of IEM. Descriptive statistical methods were used in the data analysis.

**Results:**

We included 144 patients in the study, of which 62.5% were male. The mean and median ages were, respectively, 4.3 ± 4.7 years and 2.6 years. Twelve patients (8.3%) had a confirmed diagnosis of IEM (three with aminoacidopathies, three with organic acidemias, two with urea cycle disorders and four with lysosomal storage diseases). Cognitive impairment and seizures were the initial signs and symptoms, followed by growth retardation, neuropsychomotor developmental delay, seizures and hepatomegaly. The main laboratory abnormalities in the diagnosis were hyperammonemia and metabolic acidosis.

**Conclusions:**

The diagnosis of IEM still creates challenges to the pediatric practice. In this study, we identified the following factors: difficulty to access specific laboratory tests, reduced number of experts and poor dissemination of knowledge among healthcare schools. The early diagnosis of IEM majorly impacts the treatment and prevention of sequelae and should be considered in the initial diagnostic hypotheses.

## RESUMO

**Objetivo:**

Avaliar a apresentação clínica inicial dos casos com diagnóstico confirmado de erros inatos do metabolismo (EIM) em um serviço de referência em atendimento pediátrico.

**Métodos:**

Estudo clínico, observacional, com delineamento transversal e de coleta retrospectiva em consulta ambulatorial de 2009 a 2013. Critério de inclusão: paciente encaminhado para investigação de EIM. Critério de exclusão: diagnóstico prévio de EIM. Variáveis analisadas: dados de identificação; situação atual da investigação diagnóstica; história familiar; apresentação clínica inicial; alterações laboratoriais. Os dados foram analisados por meio de estatística descritiva

**Resultados:**

Incluídos 144 pacientes, sendo 62,5% do sexo masculino. A mediana de idade foi de 2,6 anos e a média de 4,3 ± 4,7 anos. Doze pacientes (8,3%) tiveram o diagnóstico confirmado (três com aminoacidopatias, três com acidemias orgânicas, dois com distúrbios do ciclo da ureia e quatro com doenças de depósito lisossômico). Déficit cognitivo e convulsões foram os sinais e sintomas iniciais; seguidos de retardo de crescimento, atraso do desenvolvimento neuropsicomotor, convulsões e hepatomegalia. As principais alterações laboratoriais encontradas foram hiperamonemia e acidose metabólica.

**Conclusões:**

O diagnóstico dos EIM ainda traz desafios à prática pediátrica. Neste estudo foram identificados os seguintes fatores: dificuldade de acesso aos exames laboratoriais específicos, reduzido número de especialistas e pouca difusão do conhecimento nas faculdades da área da saúde. O diagnóstico precoce dos EIM tem impacto fundamental no tratamento e prevenção das sequelas, devendo ser considerado já nas hipóteses diagnósticas iniciais.

## INTRODUCTION

Inborn errors of metabolism (IEM) are disorders genetically determined by deficiencies of an enzyme involved in the synthesis, transport or degradation of molecules in a metabolic pathway. The occurrence of a block in one stage of a pathway results in the lack or excess of a particular substance and may, in addition, interfere in an alternative metabolic pathway. These diseases are rare, but not uncommon, considering the number of existing disorders: over 500 are known, which corresponds to about 10% of genetic diseases, with a cumulative incidence of 1:2,000 live births.^1^ ^,^ ^2^ In addition, the joint frequency of IEM in high-risk groups may be up to 200 times higher than that assessed in the general population. A specialized pediatrician with access to laboratory tests will reach a diagnosis of IEM in approximately 6 % of cases, according to estimations.^3^

From the standpoint of pathophysiology, IEM can be divided in three major groups:

1. disorders in the synthesis or catabolism of complex molecules, which are characterized by permanent and progressive signs and symptoms, without direct association to dietary intake or infections. They are classified into lysosomal storage diseases, peroxisomal disorders, congenital disorders of glycosylation and cholesterol alterations;
2. diseases that lead to intoxication, with acute or progressive signs and symptoms, usually with symptom-free intervals which may be related to dietary intake or metabolic stress. They are classified into aminoacidopathies, organic acidemias, urea cycle disorders, sugar intolerance, porphyria and heavy metal poisoning;
3. diseases involving energy metabolism: defects in energy production or use, which are characterized by disorders in the hepatic, muscular and cerebral intermediary metabolisms. They are classified into mitochondrial diseases and cytoplasmic energy defects;

The most frequently inheritance pattern involved is the autosomal recessive pattern, with possible X-linked recessive inheritance and, more rarely, an autosomal dominant mechanism.^3^ ^,^ ^4^

Depending on the enzymatic deficiency and metabolic disorder, the onset of symptoms can occur in the neonatal period, with lost suction, hypotonia, lethargy, vomiting and seizures, a situation often confused with infectious diseases.^5^ In other situations, IEM develops later, with a symptomatology determined by acute metabolic stress and periods of remission, if triggering factors are controlled. The evolution of the disease may be slower and include developmental delay, dysmorphia and recurrent infections.^1^

Several factors contribute to the difficulty in diagnosing IEM, such as the large number of disorders, the diversity of defects involved and the absence of specific signs and symptoms in most cases, causing these pathologies to be only considered by the pediatrician later.^3^ ^,^ ^6^ The laboratory techniques required for the diagnosis of IEM include from metabolic screenings in urine and plasma to enzyme assays in leukocytes, fibroblasts and, more rarely, molecular analysis.

The lack of epidemiological data in the Brazilian pediatric population hinders the diagnostic search.^7^ Delayed diagnosis and therapy are often associated with progressive neurological lesions and risk of death.^1^ The pediatrician must become familiar with the clinical presentation of such disorders, and with the best emergency management to stabilize seriously ill patients and identify children who can benefit from specific assessment and treatment. Recent advances in the diagnosis and treatment of IEM have significantly improved prognosis for many of these diseases.^3^ Considering the technical difficulties and costs involved, proper guidance on laboratory investigation based on the main clinical and laboratory findings is important.^3^ Therefore, this study aimed to assess the initial clinical presentation of the cases with a confirmed diagnosis of IEM at a reference facility for pediatric care.

## METHOD

We performed an observational and cross-sectional clinical study, with retrospective data collection for outpatients from 2009 to 2013. The study was designed in accordance to the regulatory guidelines and standards for research involving human beings and approved by the Research Ethics Committee of the Joana de Gusmão Children’s Hospital (*Hospital Infantil Joana de Gusmão*; HIJG), according to Resolution No. 004/2014. The HIJG is located in Florianópolis, Santa Catarina State, and is linked to the State Department of Health. It acts as a State reference facility for children with illnesses that require medium- and high-complexity research or treatment. In Genetics, HIJG stands out in the care and follow-up of patients from neonatal screening and enzyme replacement therapy for mucopolysaccharidoses.

We analyzed these patients’ medical records in search of signs, symptoms, laboratory tests, age at clinical suspicion, sex and family history. The inclusion criterion was to have been referred to the hospital for IEM investigation. The exclusion criteria were:

- Patients who received a prior diagnosis of an IEM and therefore only receive follow-up care at the hospital.
- People with phenylketonuria, cystic fibrosis, galactosemia and biotinidase deficiency, as these diseases are part of neonatal screening in Santa Catarina.

All the included patients were assessed by Genetic Services. The samples collected were sent to the IEM and Mucopolysaccharidosis (MPS) Networks, headquartered at the Hospital das Clínicas de Porto Alegre (HCPA).

The analyzed variables were divided into the following groups:

- Patient identification data.
- Initial clinical presentation.
- Family history of consanguinity and other cases of IEM in the family.
- Laboratory changes related to the IEM hypothesis.
- Status of the diagnostic investigation: inconclusive or confirmed diagnosis.

All the information was entered into a database and later analyzed with SEstatNet^®^ ^8^ (Florianópolis, Santa Catarina, Brazil). The team used descriptive statistical procedures, analyzing measures of central tendency and variation.

## RESULTS

Ninety (62.5%) of the 144 patients who met the inclusion criterion were male. The mean age of patients with clinical suspicion of IEM was 4.3 ± 4.7 years, with a median of 2.6 years, ranging from 3 days to 18 years of age (Table 1).

**Table 1:**
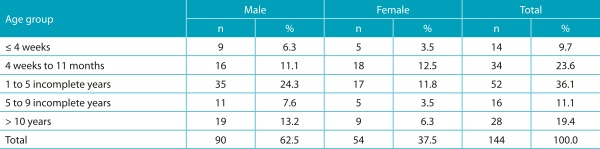
Distribution of patients with suspected inborn errors of metabolism according to age at clinical suspicion and sex, at the Joana de Gusmão Children’s Hospital, Santa Catarina, Brazil.

At the time of the study, 12 patients (8.3%) had a confirmed diagnosis of IEM, while 43 (29,8 %) remained with an inconclusive diagnosis. Cases in which new samples were requested by the reference laboratory but that, up to the time of the study, had not been sent due to loss of outpatient follow-up or death, were deemed inconclusive. The majority of the patients (61.8%) had the diagnosis of IEM discarded through specific tests. The 12 confirmed cases of IEM were summarized in the following groups:

- Aminoacidopathies (one tyrosinemia; two maple syrup urine diseases).
- Organic acidemias (one methylmalonic acidemia; two glutaric acidemia type I).
- Urea cycle disorders (one hyperinsulinism/hyperammonemia; one ornithine transcarbamylase deficiency).
- Lysosomal storage diseases (two mucopolysaccharidoses type I; one Krabbe disease, one Pompe disease).

Consanguinity was identified in 9.7% of patients, but no family history of previously diagnosed metabolic disorders was identified. The most common clinical presentation of patients referred to IEM investigation was neuropsychomotor developmental delay (NPMD), which occurred in half of the patients, followed by cognitive impairment and seizures (Table 2). When only 12 patients with a confirmed diagnosis were considered, the most important clinical data were cognitive impairment and seizures. Growth deficits and hepatomegaly were also observed in these 12 patients.

**Table 2:**
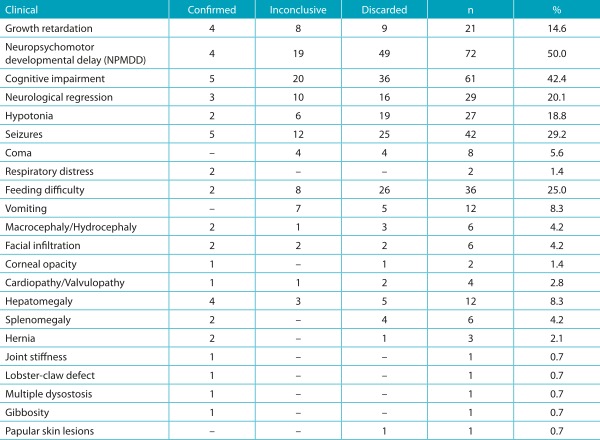
Initial clinical presentation of patients with suspected inborn errors of metabolism according to the diagnostic assessment at the moment of the study, at the Joana de Gusmão Children’s Hospital, Santa Catarina, Brazil.

*The same patient may present more than one clinical finding. NPMD: neuropsychomotor development.

Two of the three patients with aminoacidopathies presented cognitive impairment. In cases of organic acidemia, all children had seizures. Patients with urea cycle disorders had feeding difficulty, growth retardation and NPMD; no findings were striking. Among patients with lysosomal storage diseases, hepatomegaly was the most frequent finding (Table 3).

**Table 3:**
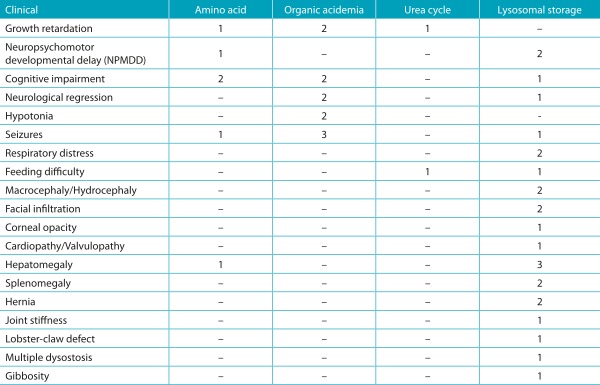
Initial clinical presentation of patients with a confirmed diagnosis of inborn errors of metabolism, at the Joana de Gusmão Children’s Hospital, Santa Catarina, Brazil.

*The same patient may present more than one clinical finding; NPMD: neuropsychomotor development.

The primary alteration in laboratory tests, among the suspected and confirmed cases of IEM, was hyperammonemia, followed by metabolic acidosis. None of the patients investigated presented lactic acidosis, hypoglycemia and ketonuria.

## DISCUSSION

The frequency of IEM found in this study (8.3% in 144 patients) is comparable to the percentages stated in the literature for selected samples based on clinical presentation.^2^ ^,^ ^3^ ^,^ ^9^ ^,^ ^10^ ^,^ ^11^ ^,^ ^12^ ^,^ ^13^Among the studies cited in Table 4, the highest prevalences of IEM were found in reference hospitals for these diseases.^3^ ^,^ ^11^ ^,^ ^12^

**Table 4:**
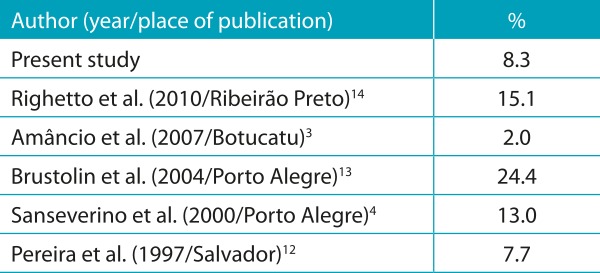
Percentage of diagnosis of IEM among suspected cases in diffrent studies.

There was a slight male predominance in both the total study sample (62.5%) and in patients with a confirmed IEM diagnosis (58.3%), which is also observed in the literature, probably due to the occurrence of IEM with X-linked recessive inheritance.^3^ ^,^ ^10^ ^,^ ^12^ ^,^ ^14^ ^,^ ^15^ ^,^ ^16^However, in the present study, this may have occurred due to the small sample size, as we only had one case of IEM with X-linked recessive inheritance - a case of ornithine transcarbamylase deficiency - in a female patient.

The mean age of patients with confirmed IEM was 4.3 years in this study, similar to what was already reported in the literature;^7^ ^,^ ^15^ which means that for some of these children with metabolic diseases, irreversible sequelae were already established. The studies by Sanseverino^4^ and Brustolin,^13^ both conducted at HCPA, showed that most patients diagnosed with IEM (80.5% and 62.5%, respectively) had symptoms before reaching 1 year of age.^3^ ^,^ ^12^ Therefore, as with the patients in this study, symptoms appeared early, but because they were non-specific, there was a delay in diagnosis. This corroborates the difficulties already mentioned in the literature, which contribute to delay the diagnosis of IEM. Such metabolic diseases are disregarded as an initial diagnostic hypothesis in case of non-specific signs and symptoms, such as vomiting, breathing difficulty, respiratory distress, developmental delay and seizures, among others.^6^ It is probable that these non-specific findings, along with the difficulty to access specific laboratory tests, are the causes for delayed diagnosis in most patients.

Characteristically, small-molecule metabolic diseases (aminoacidopathies, organic acidurias, urea cycle disorders and fatty acid oxidation disorders) are diagnosed earlier compared to classes of IEM.^14^This is because the molecules from the intermediary metabolism circulate in all or almost all body compartments, producing early and severe conditions, with marked symptoms and, in some cases, with disruptive symptoms, which may appear from the first hours to the first weeks of life. The manifestations of these diseases are typically associated with the initiation of breastfeeding, infections and nutritional changes.^6^ ^,^ ^12^ In this study, the aminoacidopathies were diagnosed at 1 month of age, demonstrating the possibility of early diagnosis when clinical suspicion is raised.

In the present sample, the group of lysosomal storage diseases is noteworthy, which was rapidly diagnosed because of its specific clinical presentation, with dysmorphias and multi-systemic involvement.^7^ ^,^ ^9^ ^,^ ^10^ ^,^ ^11^ ^,^ ^15^ ^,^ ^16^ ^,^ ^17^Although the sample did not include seriously ill patients, small-molecule metabolic diseases were also identified in the present study, in agreement with other Brazilian studies.^3^ ^,^ ^12^ ^,^ ^13^

The most common clinical data among the cases referred to investigation were chronic neurological changes (NPMD and cognitive impairment). When only 12 patients with a confirmed diagnosis were considered, the most important clinical data were cognitive impairment and seizures. In the studies of Sanseverino et al.,^4^ the acute neurological alterations caused the metabolic investigation of 47.8% of the patients and were present in all those who had a definite diagnosis of an IEM.^3^ The difference in clinical findings is because, in the present study, the sample consisted of outpatients, while those in the cited literature were from pediatric or neonatal intensive care units.

Alterations in laboratory tests suggestive of metabolic diseases were reported in a few patients with suspicion of IEM and are still less frequent among those with an already confirmed diagnosis since most of the investigations were asked of outpatient appointments in metabolically stable patients.

The additional investigation needed to diagnose IEM involves a series of sophisticated equipment and costly procedures. Therefore, the current tendency has been to establish regional reference centers and the improvement of professional specialization.^3^ ^,^ ^7^ ^,^ ^12^ Although the definitive diagnosis of a metabolic disease may involve important economic issues, costs will be reduced according to the number of ordered tests and time required for the diagnosis if performed by trained professionals. The fact that sequelae in some diseases are minimized if diagnosed early, such as the maple syrup urine disease, increasing the quality of life of patients and their relatives, is also relevant. As in many other States, there are no laboratories in Santa Catarina that specialize in such diagnoses and all the samples were sent to the IEM and MPS Networks - projects that allow a partnership to diagnose such diseases through government investments.^1^

Metabolic diseases should be considered diagnostic hypotheses in all medical specialties. Signs and symptoms are often non-specific and, if suspicions are not promptly assessed, diagnosis will be delayed, with likely negative repercussions on the symptomatic treatment and worsening of the prognosis in the short and long terms.

Despite the limitations of the study, which relied on medical records and outpatient evaluation, this is the first IEM research in the State of Santa Catarina, establishing the importance of including IEM cases in clinical discussions and reinforcing that this is not a diagnosis of exclusion, therefore it should be present at the beginning of the investigation. Additional studies with a larger sample and hospital cases are necessary to verify the frequency of IEM.

The development of new therapies specific to IEM requires that professionals be attentive to such diagnoses to allow patients’ early access to treatment. Specific recommendations for some IEM diseases are being developed to guide the medical community regarding diagnosis and management. The importance of the correct diagnosis will also be reflected in the family genetic counseling, which includes planning for future pregnancies in view of recurrence risks and the possibility of prenatal diagnosis. The introduction of the National Policy on Comprehensive Care for People with Rare Diseases in the Brazilian Unified Health System, through Administrative Rule No. 199, dated January 30, 2014 will allow greater feasibility of diagnostic procedures and treatments related to genetic diseases in the country.^18^
